# Anatomical Cadaver Study of Endolaryngeal Vascularization: Focus on the Glottis, Supraglottis, and Subglottis From the Transoral Microsurgical Point of View

**DOI:** 10.3389/fonc.2018.00138

**Published:** 2018-04-30

**Authors:** Pietro Perotti, Marco Ferrari, Nausica Montalto, Davide Lancini, Alberto Paderno, Fabiola Incandela, Barbara Buffoli, Luigi Fabrizio Rodella, Cesare Piazza

**Affiliations:** ^1^Department of Otorhinolaryngology – Head and Neck Surgery, Fondazione IRCCS, National Cancer Institute of Milan, University of Milan, Milan, Italy; ^2^Department of Otorhinolaryngology – Head and Neck Surgery, University of Brescia, Brescia, Italy; ^3^Department of Otorhinolaryngology – Head and Neck Surgery, University of Genoa, Genoa, Italy; ^4^Department of Clinical and Experimental Sciences, Section of Anatomy and Physiopathology, University of Brescia, Brescia, Italy

**Keywords:** larynx, anatomy, vascularization, transoral laser microsurgery, bleeding

## Abstract

**Introduction:**

Carbon dioxide laser coagulation during transoral laser microsurgery (TLM) for laryngeal cancer allows control of bleeding from vessels smaller than 0.5 mm. Therefore, larger arteries and veins must be carefully managed by clipping and/or monopolar cautery. The aim of this paper is to detail endolaryngeal vascular anatomy and identify areas of possible bleeding during TLM.

**Methods:**

We performed an anatomical study on a series of 11 fresh-frozen human cadavers. After injection of a bicomponent red silicone into the innominate, left common carotid, and left subclavian arteries, 22 hemilarynges were dissected, the course of the supraglottic, glottic, and subglottic vessels were traced after microdissection of the intervening structures, and their size measured at specific landmark points where such vessels are more frequently encountered during TLM.

**Results:**

Three vessels arising from the superior laryngeal artery were identified after its entry point at the level of the thyro-hyoid membrane: (1) the epiglottic artery (EA), documented in 100% of cases, a common trunk dividing into two main vessels (2) the postero-inferior artery (PIA), present in 100% of the specimens, running downward and dividing in a posterior (pPIA), and anterior (aPIA) branches (3) the antero-inferior artery (AIA), present in 95% of our specimens, running downward to the anterior commissure (AC). Two transverse anastomotic networks (TANs) connected the AIA and PIA, both parallel to the vocal muscle, one lateral (present in 100% of cases), and another medial (91% of specimens). Finally, a fourth vessel supplying the glottic plane was found to be the endolaryngeal paracommissural branch of the crico–thyroid artery (PCA), arising from the inferior laryngeal artery and emerging just below the AC, through the crico–thyroid membrane (reported in 100% of the specimens). This vessel anastomosed in 91% of cases with the AIA, through one or both of the TANs.

**Conclusion:**

The course of the endolaryngeal arteries, their relationships with adjacent structures, and size at specific landmark points have been herein described in order to provide surgeons with a map to guide them during the steep learning curve of transoral surgery of the larynx, with special emphasis given to TLM.

## Introduction

Transoral laser microsurgery (TLM) has been established as an effective option in the management of Tis-T2 and selected T3 squamous cell carcinoma (SCC) of the larynx as it allows curative resections of early-intermediate supraglottic and glottic tumors within healthy narrow-free margins, sparing uninvolved portions of the larynx and their associated sphincteric functions. However, to be reproducible and effective, TLM requires specific and long-lasting training, with a perceived steep learning curve, which exposes the patients to a variable (but potentially high) rate of complications, paralleling the increasing stage and extent of disease (1, 2). In particular, intraoperative bleeding seems to be especially difficult to predict, while early posttreatment hemorrhage represents a potentially catastrophic complication since the typical postoperative scenario is, in the vast majority of patients, without tracheostomy. Such major postoperative hemorrhagic events are variably reported in the literature to range between 0.6 and 8% according to the different T categories and subsites approached (1, 3–5).

The aim of this study was, therefore, to detail the normal anatomy of endolaryngeal vascularization in order to underline the areas at higher risk for potential intra- and postoperative bleedings, with special emphasis given to the technical characteristics of the carbon dioxide laser, which is able to coagulate vessels with a maximum diameter of 0.5 mm. However, the present anatomical data may be applicable not only to TLM but also to other emerging transoral robotic or endoscopic approaches.

## Materials and Methods

Between November and December 2016, 11 larynges were harvested at the Section of Anatomy and Physiopathology, University of Brescia, Italy from fresh-frozen cadavers (all Caucasians; 6 males, 5 females; mean age at death, 64 years; range, 51–78) provided by Medcure^®^, USA, after arterial injection of a bicomponent red silicone into the innominate, left common carotid, and left subclavian arteries. Twenty-two hemilarynges were microdissected to analyze their endolaryngeal vascularization: the superior laryngeal artery (SLA) was identified and accurately followed from its entry point at the level of the thyro-hyoid membrane to its smallest branches, by considering all the intervening anastomotic networks. The inferior laryngeal artery (ILA) was analyzed in a similar fashion.

Moreover, we measured the diameter of the main laryngeal vessels using a T444.1XLR-1 Outsider Micrometer caliper (Starrett, Athol, MA, USA) to obtain a tentative measure of their suitability for coagulation by carbon dioxide laser or other surgical tools (clips and/or monopolar cautery). These measurements were obtained at specific landmark points: (1) the epiglottic artery (EA) was measured at its emergence from the SLA (landmark ea1) and at the level of the lateral margin of the suprahyoid epiglottis where the pharyngo-epiglottic fold joins the cartilage (ea2); (2) the postero-inferior artery (PIA) was measured at its origin; (3) the posterior branch of PIA (pPIA) was measured lateral to the arytenoid body, within the thyro–crico–arytenoid space (ppia1), and within the vocal muscle lateral to the vocal process (ppia2); (4) the antero-inferior artery (AIA) was measured at the level of the false vocal fold (aia1) and glottic plane (aia2).

## Results

**Figure 1:**
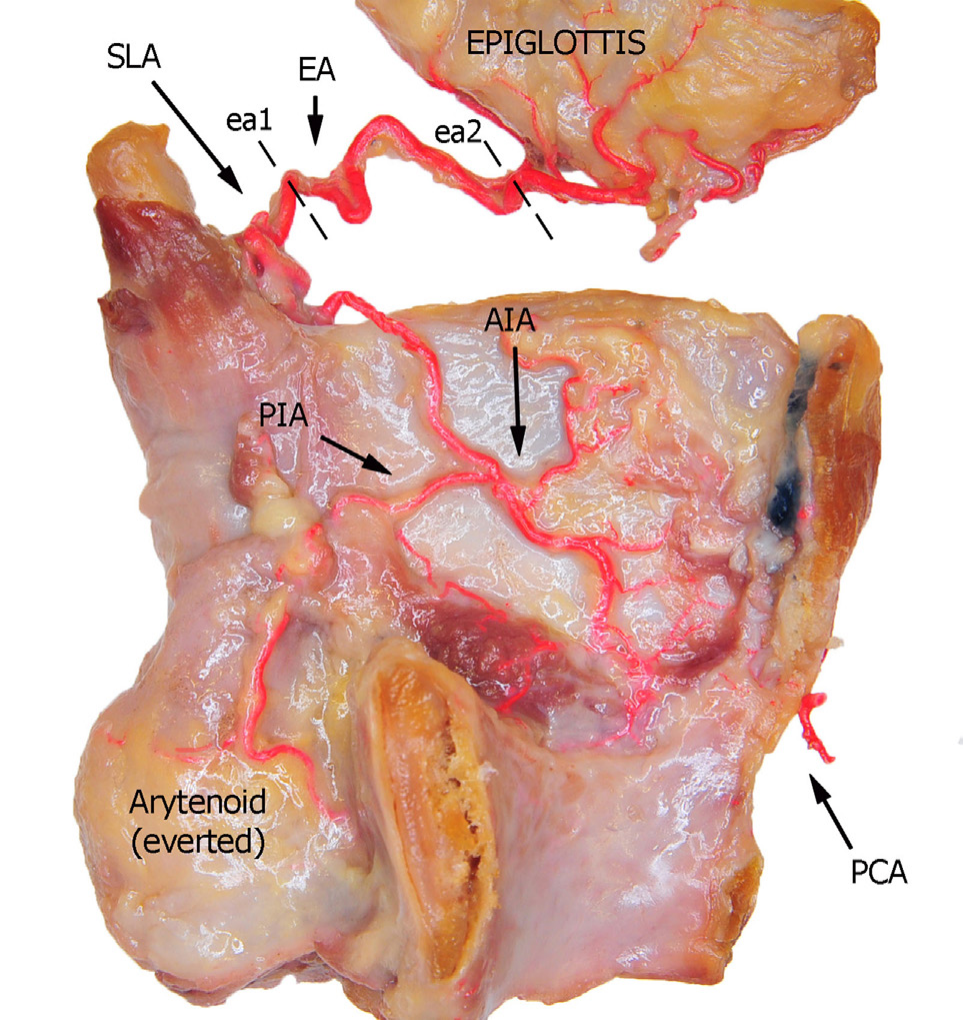
Left fresh-frozen cadaver hemilarynx after midline section of the cricoid and thyroid cartilages, eversion of the ipsilateral arytenoid, and removal of the left aryepiglottic, false, true vocal folds, and paraglottic fat tissue, thus exposing the inner thyroid lamina. Bicomponent red silicone had been injected in the left common and subclavian arteries. Dotted lines ea1 and ea2 represent the landmark points used for measurements of the EA. Abbreviations: SLA, superior laryngeal artery; EA, epiglottic artery; AIA, antero-inferior artery; PIA, postero-inferior artery; PCA, paracommissural branch of the crico–thyroid artery.

**Figure 2:**
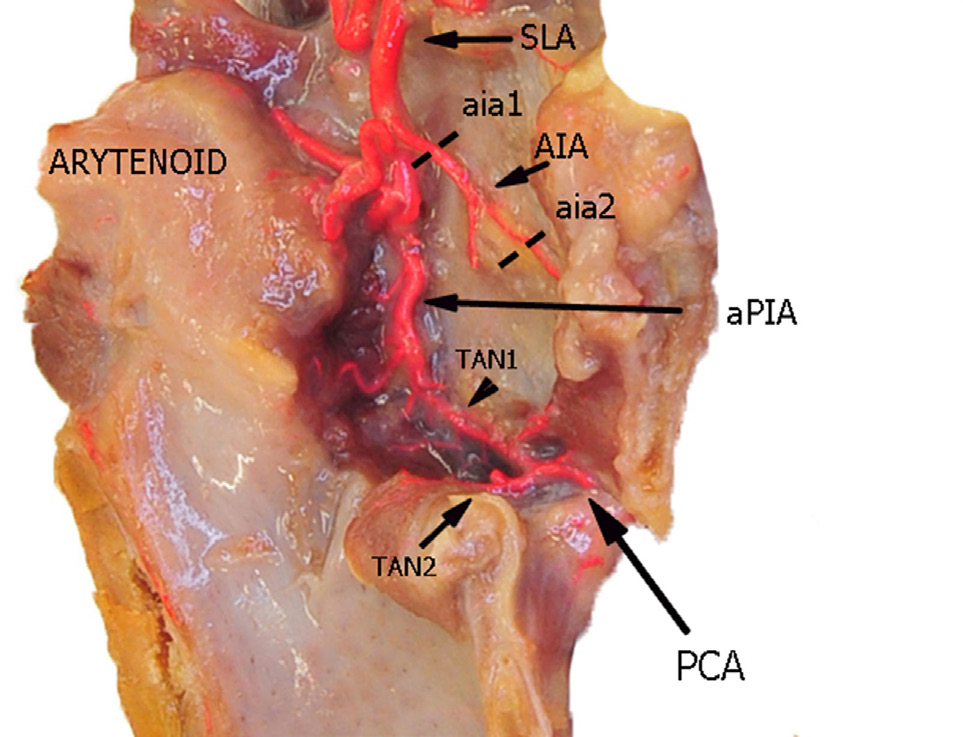
Left fresh-frozen cadaver hemilarynx after midline section of the cricoid and thyroid cartilages. The ipsilateral arytenoid was left in place, while left aryepiglottic, false, true vocal folds, and paraglottic fat tissue had been removed, thus exposing the inner thyroid lamina. Bicomponent red silicone had been injected in the left common carotid and subclavian arteries. Dotted lines aia1 and aia2 are those used for measurements of the AIA. Abbreviations: SLA, superior laryngeal artery; AIA, antero-inferior artery; aPIA, anterior branch of the postero-inferior artery; PCA, paracommissural branch of the crico–thyroid artery; TAN1, lateral transverse anastomotic network; TAN2, medial transverse anastomotic network.

**Figure 3:**
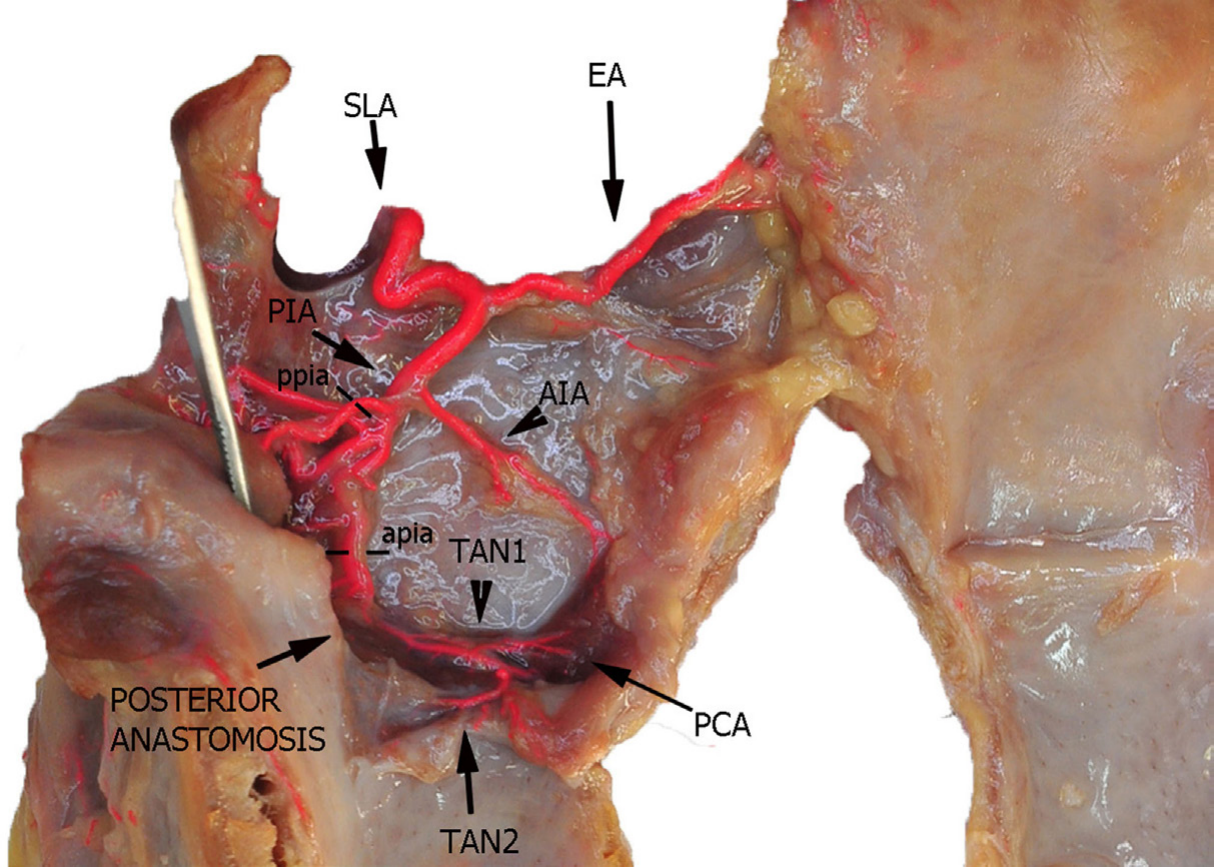
Left fresh-frozen cadaver hemilarynx after midline section of the cricoid and thyroid cartilages and partial separation of the contralateral hemilarynx. The ipsilateral arytenoid, as well as the epiglottis, was left in place and a metallic stick inserted into the piriform sinus, while left aryepiglottic, false, true vocal folds, and paraglottic fat tissue had been removed, thus exposing the inner thyroid lamina. Bicomponent red silicone had been injected in the left common carotid and subclavian arteries. Dotted line ppia was used for measurement of the posterior branch of the PIA, while dotted line apia represents the landmark used for measurement of its anterior branch. Abbreviations: SLA, superior laryngeal artery; AIA, antero-inferior artery; PIA, postero-inferior artery; PCA, paracommissural branch of the crico–thyroid artery; TAN1, lateral transverse anastomotic network; TAN2, medial transverse anastomotic network.

**Figure 4:**
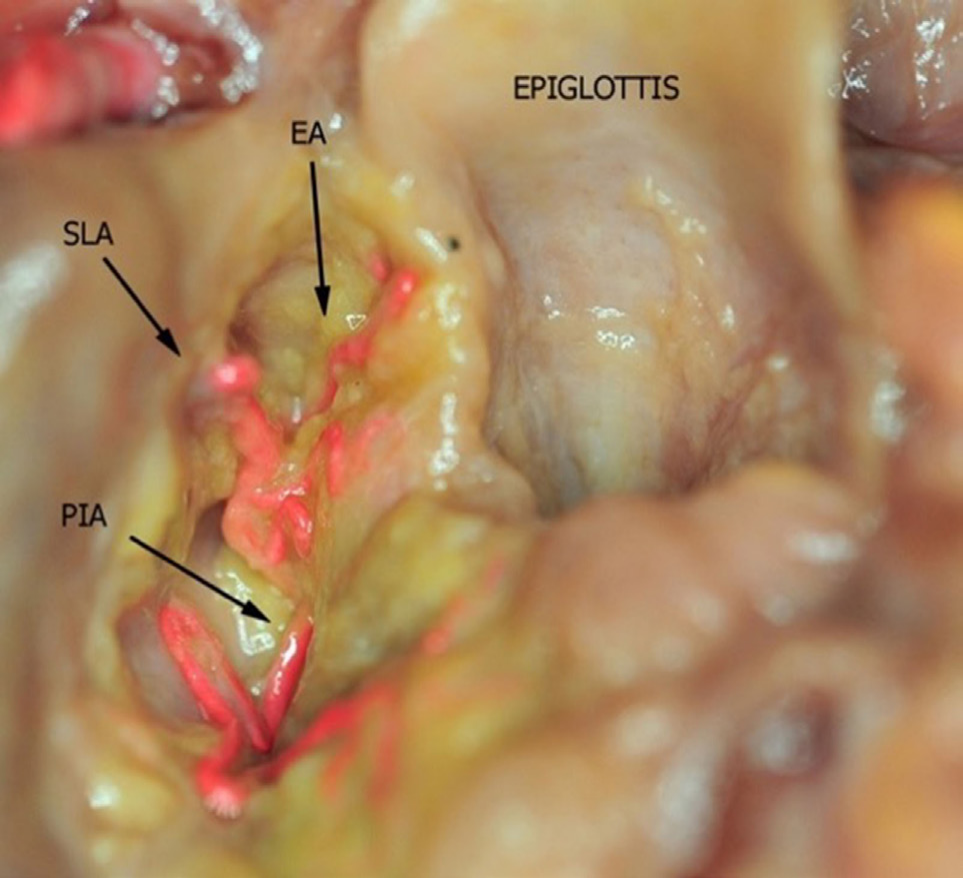
Fresh-frozen cadaver larynx seen from above after partial removal of the fat tissue within the upper and lower paraglottic space. Bicomponent red silicone had been injected in the left common carotid and subclavian arteries. Abbreviations: SLA, superior laryngeal artery; EA, epiglottic artery; PIA, postero-inferior artery.

**Figure 5:**
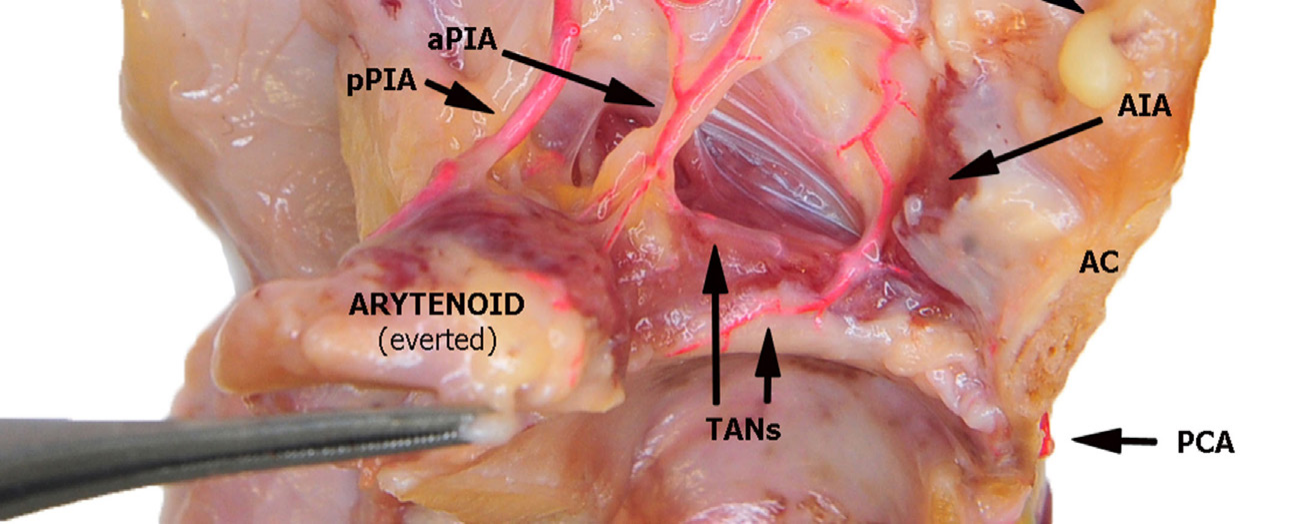
Left fresh-frozen cadaver hemilarynx after midline section of the cricoid and thyroid cartilages, seen from above. The ipsilateral arytenoid had been everted while left aryepiglottic and false vocal folds had been removed. Paraglottic fat tissue and vocal muscle are still mostly in place. Bicomponent red silicone had been injected in the left common carotid and subclavian arteries. Abbreviations: AIA, antero-inferior artery; aPIA, anterior branch of the postero-inferior artery; pPIA, posterior branch of the postero-inferior artery; AC, anterior commissure; PCA, paracommissural branch of the crico–thyroid artery; TANs, lateral and medial transverse anastomotic networks.

In the present study, three vessels arising from the SLA were identified after its entry point through the thyro-hyoid membrane. The EA was found in 100% of cases. Its caliber ranged from a median of 2.2 mm at ea1 to a median of 0.9 mm at the level of ea2 (Figures 1–3). After emerging from SLA, EA showed a common trunk with a median length of 6 mm that still divided in two main vessels: (1) the PIA, detected in 100% of the specimens (with a median diameter of 1.6 mm at its origin), run downward and divided again into two further branches, one posterior and lateral to the arytenoid (pPIA) (with a median caliber of 1.4 mm at ppia) and one directed perpendicularly to the vocal process with an antero–lateral–inferior trajectory and located within the paraglottic space (PGS) and through the vocal muscle (aPIA) (median diameter at apia of 0.9 mm) (Figures 1–5); (2) the AIA, detected in 95% of the specimens, run through the PGS toward the anterior commissure (AC) with an oblique course, and had a median caliber ranging from 1.4 mm at aia1 to 0.7 mm at aia2 (Figures 1, 2, 4 and 5).

Furthermore, two transverse anastomotic networks (TANs) connecting the AIA and PIA, both parallel to the vocal muscle, were found: one lateral (TAN1), which was identified in 100% of specimens between the muscle and the thyroid cartilage, and one medial (TAN2), which was found in 91% of specimens, constantly running between the muscle and the conus elasticus, just below the vocal ligament (Figures 2, 4 and 5). TAN1 and TAN2 were extremely variable in size and course from specimen to specimen; briefly, their caliber invariably exceeded 0.5 mm at the glottic and subglottic levels.

Finally, a fourth vessel supplying the vocal folds was found to be the endolaryngeal branch of the crico–thyroid artery, arising from the ILA and emerging just below the AC through the crico–thyroid membrane, 2–3 mm laterally to the midline. By virtue of its anatomical features, this branch was called paracommissural branch of the crico–thyroid artery (PCA). PCA was measured only outside the crico–thyroid membrane (median diameter of 1.6 mm), while it was not precisely sizeable at the level of the endolarynx (but still, a minimum diameter of 0.5 mm was observed). Identified in 100% of the specimens, this vessel anastomosed in 91% of cases with the AIA, *via* one or both TANs (Figures 1, 2, 4 and 5).

The detection rates and median sizes of the herein described vessels are reported in Table 1.

**Table 1:** Summary of each vessel’s detection rate, landmarks, and median diameter.

Vessel	Detection rate (%)	Landmarks	Median diameter (mm)
Epiglottic artery	100	ea1	2.2
		ea2	0.9

Antero-inferior artery	95	aia1	1.4
		aia2	0.7

Postero-inferior artery	100	–	1.6
pPIA	–	ppia	1.4
aPIA	–	apia	0.9
TAN1	100	–	>0.5
TAN2	91	–	>0.5
PCA	100	–	1.6

## Discussion

The first anatomical description of the endolaryngeal vascularization was published by Oki in 1958 (6). After this pioneering study, the topic was revisited by other authors (7–9), even though with an insufficient amount of details for the present purposes, and recently, with a higher resolution, on fixed larynges (10) and by angiography on fresh cadavers (11). To the best of our knowledge, microdissection on fresh-frozen specimens with the specific intention of evaluating its implications during TLM has never been accomplished before.

As it is well known, laryngeal blood supply is guaranteed by both SLA and ILA, which branch inside the organ into a number of smaller vessels whose anatomical description in terms of course, size, and possible variants is scarcely represented in the contemporary head and neck literature and not specifically finalized to TLM. Furthermore, these anatomical details are mostly overlooked even in the most prominent surgical textbooks. However, precise knowledge of the surgical anatomy of endolaryngeal vascularization is, in our opinion, of paramount importance especially when considering potentially dangerous intra- and postoperative bleedings during/after transoral procedures for laryngeal malignancies.

All the specimens of our study presented a classic course of SLA, while the variant described by Iimura et al. (10) and Rusu et al. (12) piercing the thyroid cartilage was not observed. In our experience, SLA always penetrated the thyro-hyoid membrane at a variable distance from the upper edge of the thyroid lamina, running downwards into the upper PGS (at the junction between this visceral compartment and the pre-epiglottic space) and then turning upward giving rise to the EA (also called “ascending branch of SLA” by Imanishi and coworkers) (11). The latter vessel provides blood supply to the ary-epiglottic, pharyngo-epiglottic folds, ipsilateral epiglottis, and glosso-epiglottic valleculae, where it anastomoses with the posterior branches of the lingual artery (13). Proper EA identification and its prophylactic clipping and/or monopolar cauterization are essential during TLM for supraglottic lesions. From a practical point of view, the easiest landmark for such a surgical maneuver is the pharyngo–epiglottic fold (the landmark ea1 in the present study), even though the presence of the tumor at this level may require more lateral dissection at the level of the entry point of the SLA itself.

After this first division, the common trunk of the SLA (that Imanishi et al. at this point called the “descending branch of SLA”) (11) further divides into two main branches: the PIA and AIA. The PIA usually branches in two (P1 and P2 according to the Imanishi nomenclature, aPIA and pPIA in the present study) (11), and more rarely in three vessels (18% of our series), but the areas of supply remain the same as previously described. During TLM, the most frequently encountered branch of the PIA is the aPIA, usually endangered at the level of the landmark apia during Type II or more advanced cordectomies (14), aimed at removing tissue in front of the vocal process and lateral to it in the vocal muscle. Its control is usually obtained by monopolar cauterization through insulated forceps. A more posterior resection encountering the pPIA is much rarer and mainly limited to arytenoidectomy and/or extensive aryepiglottic fold resection, where this branch may be transected at the level of our landmark ppia. Larger branches at the junction between ary- and pharyngo-epiglottic folds usually require clipping, while more distal vessels are commonly coagulated by monopolar alone.

The AIA instead runs antero-inferiorly, with terminal vessels located in the AC area, receiving anastomotic branches from the PCA, as well as with TANs. The AIA subdivisions described by Imanishi and coworkers (and named A1, A2, and A3 in that paper) (11) were inconsistently represented and identified in our study, and probably correspond to branches with a diameter inferior to the critical size of 0.5 mm. These vessels are usually encountered during Type Va and VI cordectomies at our landmark aia2 (14, 15), with the PCAs constantly found some millimeters apart from the midline, at the level of the AC. As noted, laser coagulation and/or monopolar cautery are usually sufficient to obtain bleeding control at this level. By contrast, moving downward to the anterior subcommissural level increases the risk of injuring larger branches of the crico–thyroid arteries and ILA at the level of the crico–thyroid membrane. These arteries may bleed profusely and sometimes require accurate clipping.

The TANs run parallel to the glottic plane on the two lateral (TAN1) and medial (TAN2) sides of the vocal muscle, connecting the PIA, AIA, and PCA. Even though they are usually larger than 0.5 mm, monopolar cautery is usually sufficient to control bleeding (in particular at the level of the junction between the conus elasticus and vocal ligament).

The meandering course of laryngeal vessels in specific locations (EA, PIA, and TANs) observed in the present study is consistent with that already described by Iimura and coworkers on fixed specimens (10), and related to the peculiar movements of the underlying anatomical structures during swallowing and phonation. However, we did not attempt any quantification of such a tortuous behavior of these vessels since without any practical consequence for the specific aim of our study.

**Figure 6:**
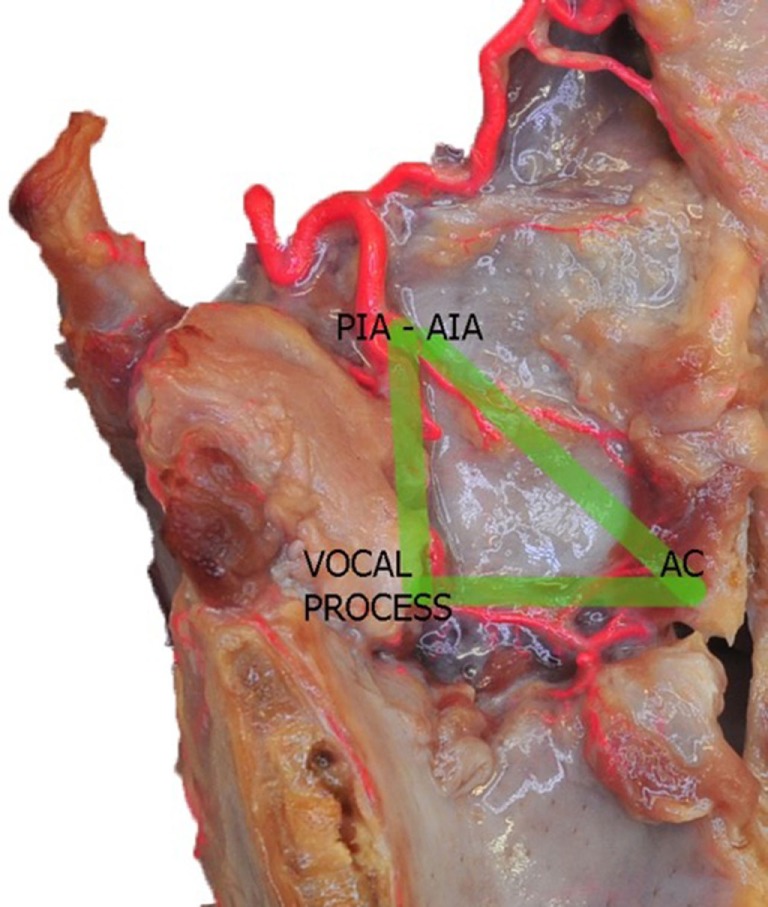
Left fresh-frozen cadaver hemilarynx after midline section of the cricoid and thyroid cartilages, and removal of the left aryepiglottic, false, true vocal folds, and paraglottic fat tissue, thus exposing the inner thyroid lamina. Bicomponent red silicone had been injected in the left common carotid and subclavian arteries. The green lines represent the “vascular” right angle triangle described in the text. Abbreviations: AIA, antero-inferior artery; PIA, postero-inferior artery; AC, anterior commissure.

In summary, two main arterial anastomotic high-flow areas can be, therefore, described in the endolarynx: (1) the AC, where branches of the AIA, PCA, and TANs join together and (2) the vocal process, where the pPIA, aPIA, terminal branches arising from the ILA, and the TANs are intermingled. If, on the sagittal plane, we trace lines connecting each other these two areas and themselves with the entry point of the SLA, we obtain a right angle triangle: the vertical side corresponds to the PIA, the horizontal to the TANs, and the oblique to the AIA, while the vertices represent the most critical areas due to their higher hemorrhagic potential (Figure 6).

**Figure 7:**
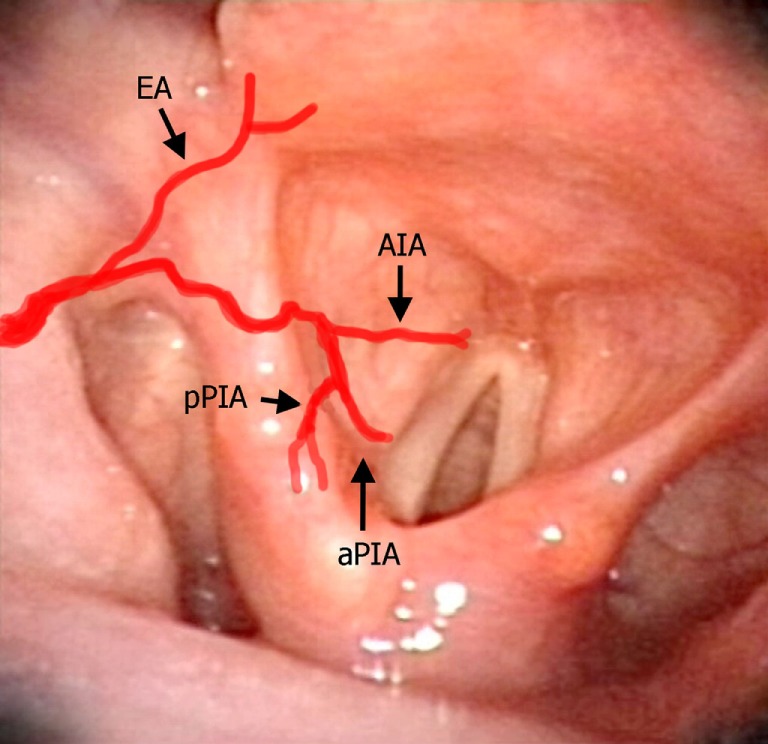
Laryngeal videoendoscopy simulating the intraoperative transoral surgical perspective, with superimposed red lines schematically representing the laryngeal arteries described in the text. Abbreviations: EA, epiglottic artery; AIA, antero-inferior artery; aPIA, anterior branch of the postero-inferior artery; pPIA, posterior branch of the postero-inferior artery.

**Figure 8:**
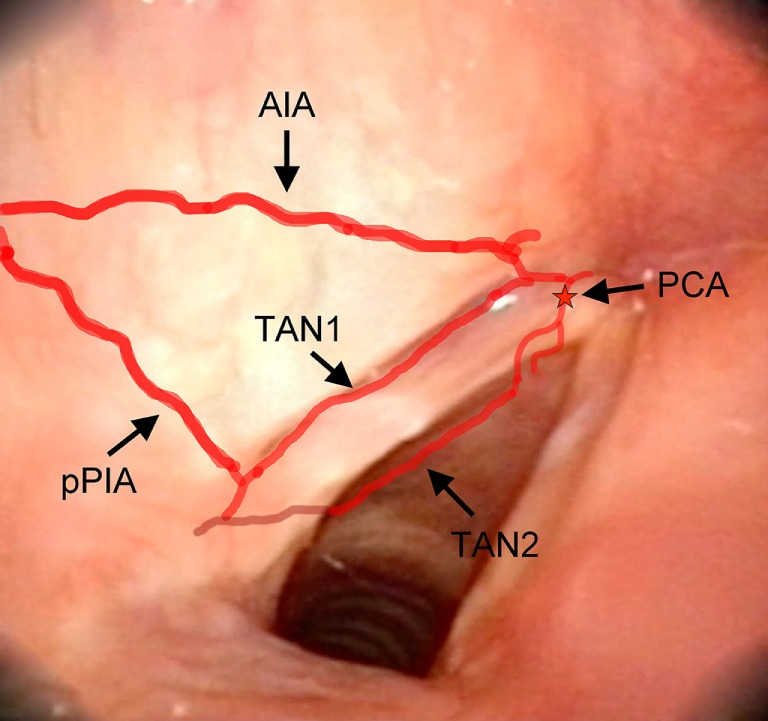
Laryngeal videoendoscopy, more closely focusing on the glottic plane. Abbreviations: AIA, antero-inferior artery; pPIA, posterior branch of the postero-inferior artery; TAN1, lateral transverse anastomotic network; TAN2, medial transverse anastomotic network; red star, endolaryngeal emergence of the PCA, paracommissural branch of the crico–thyroid artery.

Some drawbacks must be acknowledged in the present study: first, even though fresh-frozen specimens are definitely closer to normal human anatomy than fixed ones, the use of a bicomponent red silicone filling the vessel lumens may partially change their size or course. Second, use of a micrometer caliper is less precise than making cross-sectional measurements under light microscopy within histologic sections. Third, the reference to specific landmarks chosen on the basis of macroscopic laryngeal anatomy may be only in part representative of the real surgical situation and are still prone to minimal shift due to specimen distortion (cadaver larynges were no more connected to their natural fixation points in the neck). However, we believe that this level of precision is sufficient for the specific and practical purposes of the present study (Figures 7 and 8). It is nonetheless possible that a larger study on more specimens could have been more sensitive in identifying potential vascular variants and abnormalities. However, the two more recent papers in the literature similar to the present were performed on comparable (*n* = 27) (10), or smaller (*n* = 6) (11) samples. Last but not least, the present study was carried on normal larynges, while surgery for laryngeal cancer must take into account the vascular alterations induced by the presence of the tumor itself. Larger, tortuous, more fragile, and aberrant vessels are frequently encountered when dealing with SCC, especially at the level of the supraglottis. Moreover, rarer histotypes (hemangiomas, paragangliomas, sarcomas, etc.) may present with atypical vascularization requiring special caution and adjunctive measures to prevent incontrollable bleeding.

## Conclusion

The overall course, size, and distribution of the endolaryngeal vessels and their relationships with specific landmarks are described in the present anatomical cadaver study with the aim to provide surgeons with an instrument to guide them during the steep learning curve of transoral surgical approaches, in order to help in prevention and management of major intraoperative bleeding and troublesome postoperative hemorrhage.

## Author Contributions

PP, MF, NM, DL, AP, and FI: data collection and writing. BB, LR, and CP: data collection and revision.

## Conflict of Interest Statement

The authors declare that the research was conducted in the absence of any commercial or financial relationships that could be construed as a potential conflict of interest.
